# Sustainable Antibacterial Chitin Nanofiber/ZnO Nanohybrid Materials: Ex Situ and In Situ Synthesis, Characterization and Evaluation

**DOI:** 10.3390/nano15110809

**Published:** 2025-05-28

**Authors:** Caroline Piffet, Jean-Michel Thomassin, Emilie Stierlin, Job Tchoumtchoua, Claudio Fernández, Marta Mateo, Leyre Hernández, Kyriaki Marina Lyra, Aggeliki Papavasiliou, Elias Sakellis, Fotios K. Katsaros, Zili Sideratou, Dimitris Tsiourvas

**Affiliations:** 1 Celabor, Research Center, Avenue du Parc 38, 4650 Chaineux, Belgium; jean-michel.thomassin@celabor.be (J.-M.T.); emilie.stierlin@celabor.be (E.S.); job.tchoumtchoua@celabor.be (J.T.); 2 Lurederra, Área Industrial Perguita, C/A Nº 1, 31210 Los Arcos, Navarra, Spain; claudio.fernandez@lurederra.es (C.F.); marta.mateo@lurederra.es (M.M.); leyre.hernandez@lurederra.es (L.H.); 3 Institute of Nanoscience and Nanotechnology, National Center for Scientific Research ‘‘Demokritos”, 15310 Aghia Paraskevi, Greece; k.lyra@inn.demokritos.gr (K.M.L.); a.papavasiliou@inn.demokritos.gr (A.P.); or e_sakel@phys.uoa.gr (E.S.); f.katsaros@inn.demokritos.gr (F.K.K.); z.sideratou@inn.demokritos.gr (Z.S.); 4 Physics Department, Condensed Matter Physics Section, National and Kapodistrian University of Athens, Panepistimiopolis, Zografos, 15784 Athens, Greece

**Keywords:** chitin nanofibers, ZnO nanoparticles, flame spray pyrolysis, organic–inorganic nanohybrid materials, antibacterial properties

## Abstract

Diseases caused by infection are a threat to human health and the world economy, with bacterial infections being responsible for a large portion of hospitalizations, morbidity, and mortality, which necessitates the quest for advanced medications and/or sustainable antibacterial strategies. This study aims to develop bioderived chitin nanofibers (ChNFs) and ZnO nanoparticles to produce non-toxic nanohybrid materials with improved aqueous stability and enhanced antibacterial properties. These nanohybrids were formed via either (i) an ex situ route by mixing the ChNFs with ZnO nanoparticles prepared by flame spray pyrolysis or (ii) an in situ route resulting in ZnO nanoparticles being formed and embedded into ChNFs by a simple aqueous hydrothermal process, utilizing a low-cost Zn inorganic precursor. The ChNFs, the ZnO nanoparticles, and the nanohybrids were physicochemically characterized for their size, morphology, charge and stability. Their antibacterial activity was evaluated against Gram (−) *E. coli* and Gram (+) *S. aureus* bacteria, while their cytocompatibility was assessed against mammalian cell lines. The obtained results reveal a balance between antibacterial activity and cytocompatibility, as both nanohybrids exhibited satisfactory antibacterial activity (MIC 200–300 μg/mL) combined with low cytotoxicity against mammalian cell lines (cell viability 80–100%), indicating that their further application as safe and effective antibacterial agents is promising.

## 1. Introduction

Diseases caused by infection are a threat to human health and the world economy, as they are considered responsible for more than 20% of global mortality, while it is estimated that bacterial infections are responsible for about 7.7 million deaths per year, which highlights the need for sustainable and effective antibiotics [1]. Up to now, approximately 200 infectious diseases are known, while about 80% of them are spread through contaminated hands after touching contaminated surfaces [2,3]. This study aims at developing new sustainable antibacterial materials to be used as antibacterial surface coatings. In this field, interest in chitin, a naturally abundant biopolymer found in mushrooms, but also in the exoskeleton of insects and crustaceans [4], and chitin derivatives, is increasing due to their antibacterial properties and biodegradability [5,6,7,8]. Chitin and its deacetylated derivatives including chitosan demonstrate significant antibacterial properties due to their cationic nature; although there are differences between the structure of Gram (−) and Gram (+) bacteria, their effectiveness in reducing growth and multiplication is comparable [6]. However, chitin insolubility in common solvents poses restrictions in their biomedical applications, and therefore further chemical modifications or surface and bulk modifications with different antimicrobial agents, including metal oxide nanoparticles, are currently under investigation to develop new materials with improved physiochemical, biological, and ease-of-processing properties [7].

Among the inorganic-based antibacterial compounds, ZnO as well as the ZnO nanocomposites prepared employing biopolymers, such as starch, cellulose, gelatin, alginate, and poly(hydroxyalkanoates), were reported as promising candidates to be applied in eco-friendly packaging, biomedical, photocatalytic, and remediation techniques, as well as in a wide range of other utilizations [9,10,11]. Moreover, even if ZnO nanoparticles (NPs) still pose exposure risks to health associated with its concentration and the type of exposure, including contact with the skin, ingestion, and inhalation, this inorganic material in bulk form is “generally recognized as safe” and is authorized by the U.S. Food and Drug Administration [10]. However, the extensive benefits of ZnO NPs for applications in various fields, including pharmaceutical, cosmetic, textile, and agricultural, have often been limited, due to their toxicological effects on the environment and on human health [12,13,14,15]. Although the exact mechanisms by which ZnO NPs interact with living cells are still unknown, research has shown that these mechanisms are related to their interaction with proteins, DNA, and cell membranes, while ZnO NPs are implicated in the production of oxidative stress. The inherent toxicity of ZnO NPs can be effectively reduced by coating their surface with either inorganic, e.g., silica, or organic polymeric compounds, e.g., poly(sodium 4-styrenesulfonate) sodium salt, poly(allylamine hydrochloride), poly(ethylene glycol), affording novel composites or hybrid materials [16,17,18,19]. This strategy opens up the possibility for not only reducing but also improving their cellular compatibility [20].

In this study, the production of deacetylated chitin nanofibers (ChNFs) from shrimp shell wastes for the preparation of hybrid organic–inorganic-based materials was pursued, for the development of antimicrobial hybrid nanomaterials suitable for surface antibacterial coatings. Chitin nanofibers have been investigated in various areas, such as filtration, the recovery of metal ions, energy storage, cosmetics, and in biomedical fields including drug delivery, tissue engineering, and medical implants [21]. Chitin and deacetylated chitin nanofiber coatings inhibit bacterial growth, while their antibacterial properties were found to depend strongly on pH and deacetylation degree [22,23]. In our work, deacetylated chitin nanofibers, ChNFs, are envisaged to (i) function as a dispersing agent to help the stabilization of the inorganic NPs (avoid agglomeration) [24]; (ii) enhance the antibacterial properties of ZnO [25]; (iii) decrease the toxicity of the ZnO NPs [26,27]; and, if used in coating applications, (iv) to favor and increase the adhesion to the substrate thanks to covalent or ionic interactions [28,29]. In order to achieve their aforementioned roles, the ChNFs need to be chemically modified with specific groups. In the current study, the chemical modification was achieved by the introduction of amino groups in chitin chains via a simple deacetylation method.

Although the synergistic effect of ZnO NPs and ChNFs has been previously demonstrated in the literature [25], the present study goes one step further by developing and comparing the properties of ex situ and in situ formed nanohybrid materials, and finally comparing their antibacterial properties as well as their toxicities. For the development of chitin/ex situ ZnO nanohybrids, ZnO NPs were prepared by flame spray pyrolysis (FSP) and allowed to interact with chitin nanofibers prepared from raw chitin after chemical/mechanical treatment [30,31]. On the other hand, for the development of chitin/in situ ZnO nanohybrids, a low-cost and effective hydrothermal technique was applied for the in situ preparation of ZnO nanoparticles in the presence of chitin nanofibers. The obtained hybrid nanomaterials were physicochemically characterized and evaluated regarding their antibacterial properties against Gram (−) *Escherichia coli* (*E. coli)* and Gram (+) *Staphylococcus Aureus* (*S. aureus*) bacteria. Their bacteriostatic and bactericidal activity was compared to that of ZnO NPs, while in vitro cytotoxicity studies against mammalian cell lines were also performed to demonstrate their non-toxicity at antibacterial-related concentrations.

## 2. Materials and Methods

### 2.1. Chemicals and Reagents

Chitin from shrimp shells was obtained from Glentham Life Sciences Ltd. (Corsham, UK), while NaOH was purchased by VWR International BV (Leuven, Belgium). Methane and oxygen were purchased from Messer. Zinc(II) nitrate hexahydrate (≥99%), 2′,7′-dichlorodihydrofluorescein diacetate (H_2_DCFDA), ethanol, and isopropanol were obtained from Sigma-Aldrich Ltd. (Poole, UK). Dulbecco’s Modified Eagle Medium (DMEM) low glucose with phenol red, phosphate-buffered saline (PBS), fetal bovine serum (FBS), penicillin/streptomycin, L-glutamine, and trypsin/EDTA were obtained from Biowest (Nuaillé, France). Peptone from Casein was purchased from AppliChem GmbH (Darmstadt, Germany). Agar and Luria–Bertani broth (LB) were obtained from MP Biomedicals (Illkirch, France).

### 2.2. ChNFs Preparation

**Figure 1:**
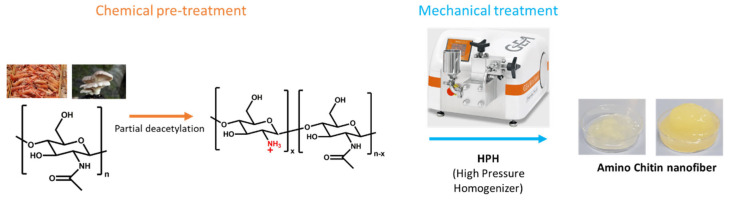
General schematic illustration depicting the preparation of chitin nanofibers.

The ChNFs were produced according to the general process showed in Figure 1. The raw chitin was first chemically pretreated and then a mechanical defibrillation was applied as exemplified in Figure 1.

#### 2.2.1. Chitin Deacetylation

The deacetylation step consisted of a chemical treatment of the chitin. In a typical reaction, chitin was added to an aqueous NaOH solution (8 mol/L) at a 4 wt. % concentration for 6 h at 90 °C. The deacetylated chitin was then rinsed several times to remove the excess of NaOH and finally collected by filtration. The as-prepared material was stored at 5 °C until the defibrillation process.

#### 2.2.2. Chitin Defibrillation

The deacetylated chitin was mechanically treated to obtain nanofibers (NFs). The defibrillation step was performed by five passages in a GEA Lab High-Pressure Homogenizer PandaPLUS 2000 (HPH) (GEA, Dusseldorf, Germany) at 0.5 wt. % in deionized water.

### 2.3. ChNFs Characterization

The deacetylation degree (DDA) of the chitin was quantified by FTIR spectrometer (Thermoscientific Nicolet iZ10, Waltham, MA, USA) according to the method of Moore and Roberts as described in the literature [32], $DDA=100-\frac{A_{1655}}{A_{3450}}\times \frac{100}{1.33}$, where A_1655_ and A_3450_ are the absorbance at 1655 cm^−1^ and 3450 cm^−1^, respectively.

In order to verify that the employed chemical/mechanical method resulted in successful defibrillation and fiber size uniformity of ChNFs dispersions, prior to their use in the following experiments, we proceeded with the preparation and optical characterization of a chitin nanofibers cast film. Thus, after the defibrillation step, a film was prepared by mixing 80 g of a 0.5 wt. % ChNFs dispersion with 0.4 g of pure glycerol and 25 g of water. This solution was placed in a 10 cm Teflon mold and dried at 60 °C. The thickness of the obtained film was about 40 ± 1 µm, as measured by a Büchel micrometer. The transmittance of the film was measured in the spectral range from 200 to 800 nm, employing a UV–vis spectrometer (GENESYS™ 150, Thermo Fisher Scientific, Inc., Madison, WI, USA).

Transmission electron micrographs of ChNFs were obtained on a FEI Talos F200i field-emission (scanning) transmission electron microscope (Thermo Fisher Scientific Inc., Waltham, MA, USA) operating at 200 kV coupled with a 100 mm^2^ X-Flash 6|T windowless energy-dispersive spectroscopy microanalyzer (Bruker, Hamburg, Germany). A drop of an aqueous dispersion (0.01 wt. %) was cast on a TEM grid and allowed to evaporate.

The viscosity of the ChNFs aqueous suspensions were determined on a Brookfield DV-I viscosimeter (Brookfield Engineering Laboratories, Inc.; Middleboro, MA, USA) at 50 rpm. The *ζ*-potential values were measured employing a ZetaPlus instrument (Brookhaven Instruments Corp, Long Island, NY, USA). For these experiments, aqueous ChNFs dispersions (0.25 wt. %), at pH values ranging between 2 and 10, were used. For each dispersion, ten measurements were collected and the results were averaged.

### 2.4. ZnO NPs Preparation and Characterization

#### 2.4.1. ZnO NPs Preparation

ZnO nanoparticles were produced following the one-step liquid flame spray pyrolysis (FSP) process, in a prototype reactor constructed by the Technological Centre Lurederra with a production capacity of 100 g of pure nanopowder per hour (Figure S1a). The liquid precursor mixture consisted of a homogeneous zinc nitrate solution in ethanol with a final zinc concentration in the range of 1.2–1.4 mol/L. The optimum calorimetric characteristics of the ZnO precursor mixture for the FSP process were 4923 cal/g and 5.6 cPs of viscosity. The precursor mixture was fed into the equipment at a flow rate of 10–30 mL/min. Oxygen was selected as a dispersion gas and set at 20–30 mL/min, while the ratio of methane to oxygen gas flow rate was 3–6 L/h to form a self-maintaining main core flame (Figure S1b). The nozzle pressure was typically around 1.5–3 bar. Finally, the obtained ZnO NPs in powder form (Figure S1c) were immediately collected in a specific sleeve filter system. Further, the ZnO NPs were dispersed in water using sonication and stirring to obtain homogeneous dispersions for the ex situ ZnO nanohybrid preparation as shown in Figure S1d,e.

#### 2.4.2. ZnO NPs Characterization

The specific surface area of the ZnO nanoparticles was determined employing by nitrogen adsorption at −196 °C, in a Tristar II 3020 multi-sample specific surface analyzer (Iberfluid, Madrid, Spain) using the BET method. Transmission electron microscopy (TEM) images were obtained using a JEOL, Mod. JEM-2010 (JEOL, Tokyo, Japan). The X-ray diffraction (XRD) patterns were recorded using a Nano-inXider from Xenocs SAS (Grenoble, France) equipped with a Xenocs GeniX3D Cu Kα source (1.54189 Å wavelength) and two 2-D Pilatus X-ray detectors for simultaneous SAXS/Wide angle X-ray scattering (WAXS) measurements, without beam stop. The samples were mounted into stainless-steel washers (0.5 mm height), capped with Kapton tape. The acquisition time was set to 3600 s at medium resolution. The q range was recorded from 0.0026 to 4.20 Å^−1^. The built-in XSACT software version 2.10.3 was applied for the background subtraction and the 1D merging of the spectra. Particle size distribution was determined at 25 °C from dynamic light-scattering experiments by employing a Z-sizer equipment (Zetasizer Nano Series, Malvern Instruments, Malvern, UK). For these measurements, a 0.01 wt. % ZnO NPs aqueous dispersion was utilized. Finally, the ζ-potential values were measured employing the ZetaPlus instrument (Brookhaven Instruments Corp, Long Island, NY, USA) as described above. For these experiments, aqueous dispersions (0.05 wt. %), at pH values ranging between 2 and 10, were used.

### 2.5. ChNFs/Ex Situ and In Situ ZnO Nanohybrid Preparation and Characterization

#### 2.5.1. ChNFs/Ex Situ ZnO Nanohybrid Preparation

**Figure 2:**
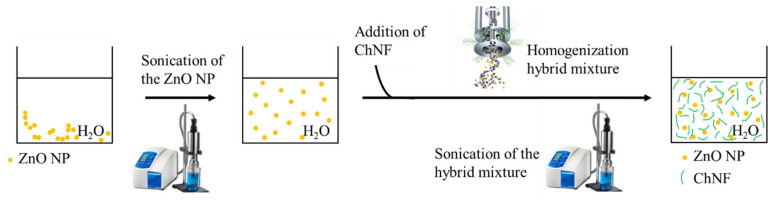
Procedure for the preparation of the ChNFs/ex situ ZnO nanohybrid.

The hybrid mixtures of ZnO NPs + ChNFs were prepared by the procedure illustrated in Figure 2. The preformed ZnO NPs were properly dispersed in water (0.1 wt. %) thanks to a sonication step with a Branson SFX250 sonifier (Danbury, CT, USA). Then, an equal volume of a water and 0.5 wt. % dispersion of ChNFs was added. The mixture was firstly homogenized with a laboratory mixer Silverson L5M-A and then an additional sonication step was performed. For 50 mL of hybrid mixture dispersions, the amplitude was set at 50%, pulsation at 0.5 s and time at 1 min, employing a 12 mm probe. Thus, the final concentration of the mixture was 0.05 wt. % of ZnO NPs and 0.25 wt. % of ChNFs.

#### 2.5.2. ChNFs/In Situ ZnO Nanohybrid Preparation

Dispersions of chitin nanofibers were used as templates for the in situ synthesis of chitin nanofibers/ZnO hybrid (ChNFs/in situ ZnO). Specifically, this nanohybrid material was prepared in the presence of chitin nanofiber dispersion by a hydrothermal method in alkaline medium at 70 °C. Briefly, a solution of zinc(II) nitrate hexahydrate (0.2 g in 4 mL water) was added to a 0.5 wt. % dispersion of chitin nanofibers in water (50 g) under continuous mixing and to the resulting mixture was added NaOH solution until the pH of the final dispersion increased to 12. The mixture was allowed at 70 °C for 24 h under continuous stirring. The resulting dispersion was centrifuged, and the obtained material was repeatedly redispersed in water and centrifuged 3 times to remove any unreacted molecules/ions. The final ChNFs/in situ ZnO nanohybrid material was preserved as a dispersion to be used for antibacterial and cytotoxicity evaluation experiments, while ChNFs/in situ ZnO was also obtained as a powder after lyophilization to be used in characterization experiments (FTIR and XRD). Quantitative determination of the polymer content in the final hybrid nanoparticles was achieved by determining the weight loss after heating the materials up to 800 °C at a heating rate of 5 °C/min under air atmosphere. The absence of any carbonaceous mass was confirmed visually by the final clear white color in the residues. It should also be noted that regarding the original ChNFs, under these experimental conditions, no residual weight was obtained.

#### 2.5.3. ChNFs/Ex Situ and In Situ ZnO Nanohybrid Physicochemical Characterization

Transmission electron micrographs were acquired using a FEI Talos F200i field-emission (scanning) transmission electron microscope (Thermo Fisher Scientific Inc., Waltham, MA, USA) as described above. FTIR spectra were registered on a Nicolet 6700 spectrometer (Thermo Scientific, Waltham, MA, USA) coupled with a Specac Quest ATR with a diamond crystal (Specac Ltd., London, UK) at 4 cm^−1^ resolution. The *ζ*-potential values were measured employing a ZetaPlus instrument at pH values ranging between 2 and 10, as described above. For these experiments, aqueous dispersions of ChNFs/ex situ ZnO (0.25 wt. % ChNFs + 0.05 wt. % ZnO NPs) and ChNFs/in situ ZnO (0.03 wt. %) were used. Dynamic light scattering (DLS) studies of the above aqueous dispersions of ChNFs/ex situ and in situ ZnO nanohybrids as well as of the parent chitin nanofibers (0.25 wt. %) were performed employing an AXIOS-150/EX (Triton Hellas, Thessaloniki, Greece) apparatus with a 30 mW laser source and an Avalanche photodiode detector at an angle of 90◦. The autocorrelation functions were analyzed using the CONTIN algorithm to obtain the apparent hydrodynamic radii distributions.

#### 2.5.4. ChNFs/Ex Situ and In Situ ZnO Nanohybrid Antibacterial Evaluation

*Bacterial strains*: Gram (−) *Escherichia coli* strain DH5*α* (*E. coli*) and Gram (+) *Staphylococcus aureus* strain ATCC 25923 (*S. aureus*) were used as model microorganisms for the antibacterial activity evaluation of the developed ZnO-NFs hybrids as well as of the parent ZnO NPs and ChNFs, according to the CLSI guidelines (CLSI documents M26-A and M07-A9 [33,34]. *E. coli* bacteria were grown in Luria–Bertani (LB) medium for 18 h, while *S. aureus* bacteria were grown in tryptic soy broth (TSB) medium for 16 h. *E. coli* bacteria were incubated at 35 °C under a relative humidity of about 90 %, while *S. aureus* were grown in aerobic conditions at 37 °C in a Stuart SI500 orbital shaker (~200 rpm rotation speed, Bibby Scientific Ltd, Staffordshire, UK). Bacterial inoculums with a turbidity equal to that of 0.5 McFarland standard were prepared and used for the experiments that followed. The turbidity *E. coli* and *S. aureus* bacteria suspensions was recorded at 600 nm, employing a Helios gamma spectrophotometer (Thermo Fisher Scientific Inc., Waltham, USA) and a Cary 100 Conc UV–visible spectrophotometer (Varian Inc., Mulgrave, Australia), respectively.

*Minimum inhibitory concentrations (MIC):* For the determination of MIC values of both ZnO nanohybrids as well as of the parent ZnO NPs and ChNFs, the broth macro-dilution method was employed, following the M07-A9 protocol, published by the Clinical Laboratory Standards Institute (CLSI) [34]. Briefly, the bacteria subcultures were diluted to a final concentration of 5 × 10^5^ CFU/mL, while serial two-fold dilutions of materials in growth media (LB or TSB) were performed to obtain dispersions at various concentrations ranging from 5 μg/mL to 500 μg/mL. Then, each dispersion was added to an equal volume of bacteria suspension (2 mL total volume) at 35 °C (*E. coli*) or 37 °C (*S. aureus*). Untreated bacteria or growth media without bacteria were used as positive and negative controls, respectively. After a 24 h incubation time, MIC was determined as the lowest concentration at which no visible bacterial growth was observed.

*Minimum bactericidal concentration (MBC):* For the determination of MBC values of all compounds, the colony counting method were employed, following the M26-A protocol, published by the Clinical Laboratory Standards Institute (CLSI) [33]. Briefly, aliquots of 100 μL from the tubes at MIC value and three tubes above the MIC value that did not show visible bacteria growth were diluted, and each dilution was spread on agar plates. After a 24 h incubation time, the colonies of bacteria (CFU/mL) were counted, and the reduction in bacterial growth was calculated for every sample. MBC was determined as the lowest concentration in which the growth of the initial bacterial inoculum was inhibited by 99.9%.

*Intracellular reactive oxygen species (ROS) generation*: The reactive oxygen species (ROS) production in bacteria upon treatment with ZnO NPs or ZnO nanohybrids were measured using 2′,7′-dichlorodihydrofluorescein diacetate (H_2_DCFDA), a well-known dye used for determination of the ROS level or redox state of the cells, following the fluorometric method described in the literature [35,36]. In brief, bacteria (1 × 10^8^ CFU/mL) were treated with 10, 20, or 30 μg/mL ZnO NPs, 60, 120, or 180 μg/mL ChNFs/ex situ ZnO (corresponding to 10, 20 or 30 μg/mL ZnO, respectively) or 28.5, 57, or 85.5 μg/mL (corresponding to 10, 20 or 30 μg/mL ZnO, respectively) ChNFs/in situ ZnO at 37 °C in a Stuart SI500 orbital shaker (~200 rpm shaking speed). Untreated bacteria were used as control. After a 5 h incubation time, 10 μM H_2_DCFDA was added to each tube and incubated in the dark at 30 °C for 45 min. Then, the bacterial suspensions were centrifuged, washed, resuspended in PBS, disrupted by ultrasonication and finally centrifuged once more. Fluorescence intensity of the supernatant was measured (λ_ex_ = 485 nm; λ_em_ = 530 nm) with an Infinite M200 microplate reader (Tecan group Ltd., Männedorf, Switzerland).

*Bacteria morphological study*: The morphology of bacteria after treatment with either ChNFs/in situ ZnO or ChNFs/ex situ ZnO hybrids was studied by scanning electron microscope (SEM). Briefly, the cells were exposed to ChNFs/in situ ZnO or ChNFs/ex situ ZnO hybrids at a concentration equal to half of the minimum inhibitory concentration (½MIC) for 12 h. Subsequently, *S. aureus* bacteria were treated with a glutaraldehyde solution (3 wt.%) in sodium cacodylate buffer (100 mM, pH = 7.1) for 12 h. Afterwards, the cells were collected and then washed to remove the excess of glutaraldehyde or any other by-products. Finally, they were re-suspended in the same buffer and 50 μL of each cell dispersion was deposited onto a glass cover slip coated with poly(l-lysine). The samples were then dehydrated using a series of ethanol concentrations (50%, 70%, 95%, and 100%) for 10 min each. After drying, the samples were coated with gold using a sputter coater [37,38].

#### 2.5.5. In Vitro Cytotoxicity Studies

Human embryonic kidney HEK293 cell line, and human prostate cancer cells DU145 and PC3 were cultured in RPMI supplemented with 10% FBS and penicillin/streptomycin solution. The cytotoxicity of ZnO NPs, ChNFs, ChNFs/ex situ ZnO, and ChNFs/in situ ZnO was assessed by using the standard MTT assay. In brief, cells were grown at 37 °C in a humidified atmosphere containing 5% CO_2_ and sub-cultured twice a week after detaching with a trypsin/EDTA solution. Then, cells were inoculated (10^4^ cells/well) in 96-well plates and incubated in complete RPMI supplemented with 10% FBS for 24 h. After the incubation time, cells were treated with the tested materials at various concentrations for 24 h. Subsequently, RPMI medium was replaced with 100 μL of MTT solution (10 μg/mL in complete RPMI medium) and incubating at 37 °C in a 5% CO_2_ humidified atmosphere for 4 h. Then, formazan crystals were produced and solubilized in 2-isopropanol (100 μL/well). Infinite M200 microplate reader (Tecan group Ltd., Männedorf, Switzerland) was used to record the absorbance at 540 nm. For each concentration, six replicates were performed, while each experiment was repeated three times. Cell viability percentage was determined and compared to the control (cells incubated only with complete medium). Blank values measured in wells with 2-isopropanol and no cells, were in all cases subtracted.

To study the statistical significance of a difference between means, a Student’s paired two-tailed *t*-test was performed on the MTT cytotoxicity data obtained for all materials in comparison with control (untreated cells). The statistical significance follows the assignment: * *p* < 0.05, ** *p* < 0.01, *** *p* < 0.001, **** *p* < 0.0001, and ns, not significant (*p* > 0.05).

## 3. Results and Discussion

### 3.1. ChNFs

During the deacetylation of the chitin, a part of the acetyl groups of the chitin reacted with NaOH and were transformed to primary amino groups (Figure S2a). The deacetylation degree (DDA) of the chitin was quantified by FTIR spectroscopy (Figure S2b) as described in the Materials and Methods section. It was found that the chitin used in this study has a DDA equal to 30%. According to Boonmahitthisud et al. [39], this DDA is not only an ideal compromise for the stabilization of the chitin NFs in water, but also for the preparation of the hybrid suspensions. Indeed, the authors reported that the *ζ*-potential increased up to 30% of DDA, but then a gradual decrease was observed, which was linked to the aggregation of chitosan molecules due to inter/intramolecular hydrogen bonding. It should be noted that according to the literature, a further increase in DDA to around 40–50%, results in the transformation of chitin to chitosan, which is soluble in acidic aqueous environment [40,41].

In addition, due to the new amino groups obtained by the chemical modification step, the partially deacetylated chitin nanofibers are positively charged at a pH below the pKa of these amino groups (ca. 6-6.5). This is coherent to what is observed with the *ζ*-potential measurements (see Section 3.2). The positive charge of the nanofibers has a double role: (i) to facilitate the mechanical defibrillation with the HPH due to the electrostatic repulsion between the fibers, and (ii) to improve the interactions and stabilization of the different compounds in the hybrid suspensions.

**Figure 3:**
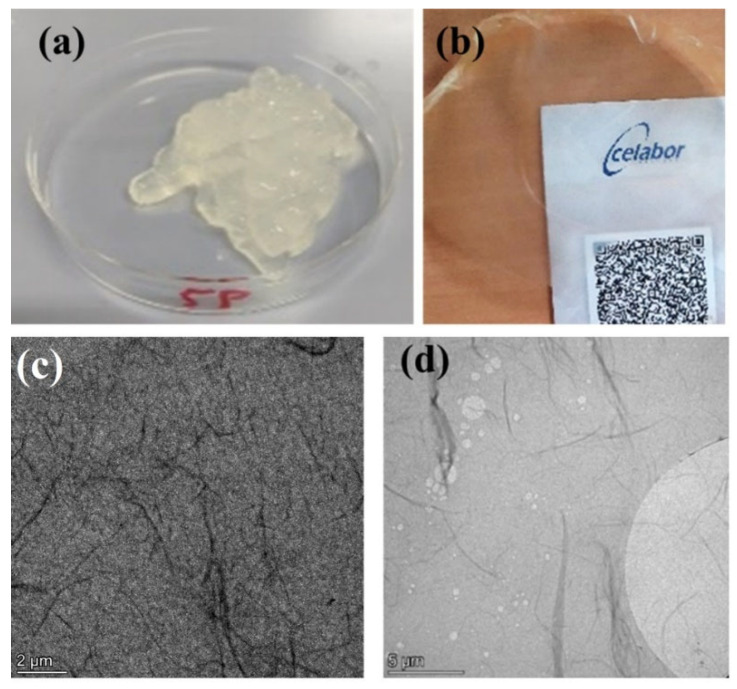
(**a**) Defibrillated chitin 0.5 wt. % in water, (**b**) film of defibrillated chitin, and (**c**,**d**) transmission electron microscopy images of ChNFs.

After the defibrillation step, a suspension of ChNFs was obtained as a gel (Figure 3a), with a measured viscosity equal to 2800 cP at 50 rpm. This very high viscosity value is in line with the literature [4]. The preparation of ChNFs film and the registering of its UV–vis spectrum was performed to provide information on the quality of defibrillation. This type of UV–visible analysis is commonly used to characterize bio-based nanofibers such as chitin and cellulose [42,43]. The light transmission of a film of defibrillated chitin with a thickness of 40 μm (Figure 3b) was about 72% in the whole 400–800 nm region (see UV–vis spectrum in Figure S2c), which confirmed the high efficiency of the defibrillation step, as it is known that the thinner and the shorter the fibers are, the higher the transparency [44]. Moreover, this is close to the values reported in a previous study on ChNFs [45]. TEM images (Figure 3c,d) clearly show the presence of nanofibers with lengths of 2–6 μm, as well as bundles of nanofibers with widths in the range of 30-80 nm. On the other hand, dynamic light scattering (DLS) experiments of 0.25 wt. % water dispersions reveal the presence of aggregates with mean radii of ca. 1000 nm (Figure S3).

### 3.2. Synthesis and Physicochemical Characterization of ChNFs/ZnO Hybrid Nanomaterials

#### 3.2.1. ChNFs/In Situ ZnO Nanohybrid

**Figure 4:**
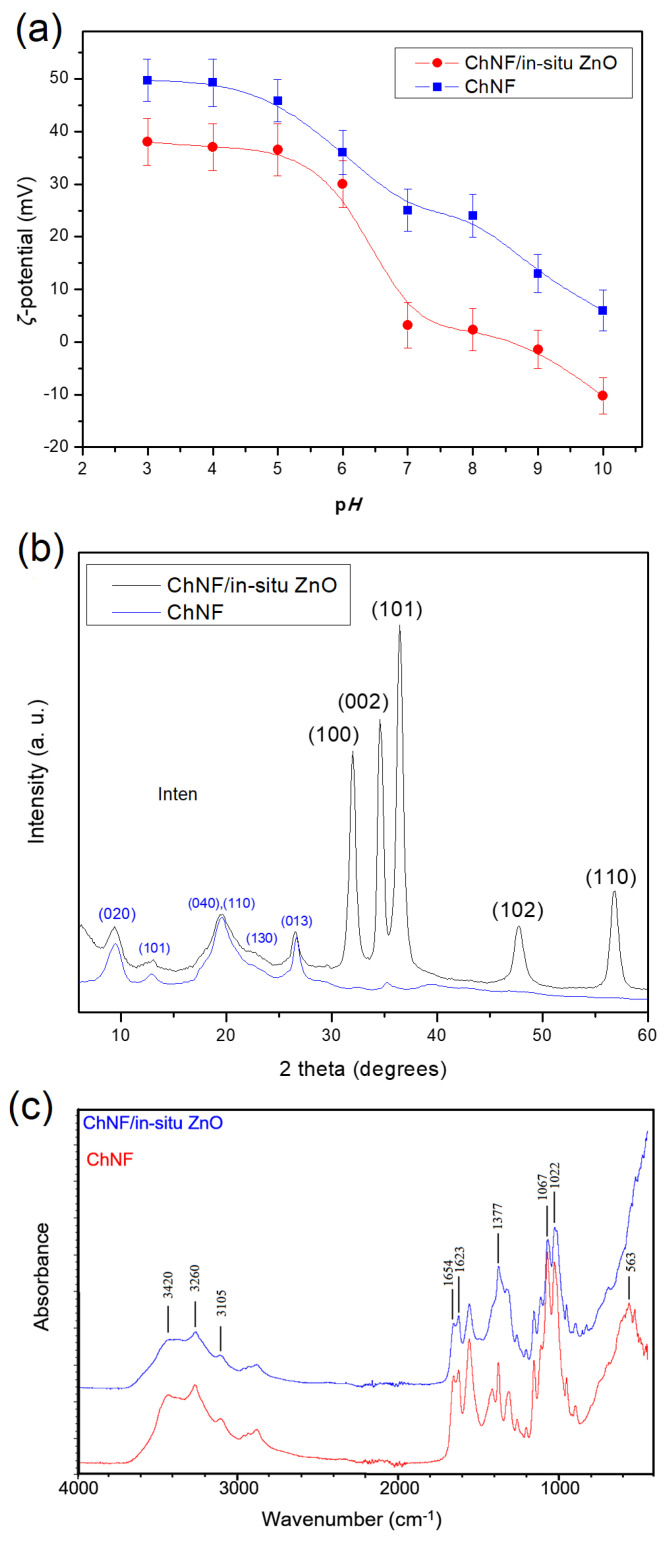
Chitin nanofibers (ChNFs) and ChNFs/in situ ZnO hybrid: (**a**) ζ-potential as a function of pH, (**b**) XRD patterns and (**c**) FTIR spectra.

The synthesis of ChNFs/in situ ZnO nanoparticles was realized following a hydrothermal method by employing a dispersion of chitin nanofibers as a template and zinc nitrate as a zinc precursor (cf. Section 2.5.2). *ζ*-potential measurements of the resulting dispersion as a function of pH value (Figure 4a), show that the presence of the negatively charged ZnO particles leads to the lowering of the positive *ζ*-potential values of the original nanofibers and also corroborate their stability at pH values below 6; instead, at pH values close to neutral, the *ζ*-potential values are close to zero, in line with the observations that under these conditions the dispersions are no longer stable. DLS measurements of ChNFs/in situ ZnO nanohybrid dispersions at neutral pHs reveal the formation of aggregates of about 1300 nm radii (Figure S4).

The obtained nanohybrid dispersion was lyophilized in order to be physicochemically characterized by FTIR spectroscopy, XRD, and TEM. The ZnO content in the dry nanohybrid was found to be 36.0 wt. % as obtained by determining the weight loss after heating the material up to 800 °C.

The X-ray patterns of both the parent ChNFs and the ChNFs/in situ ZnO hybrid after lyophilization are shown in Figure 4b. For the ChNFs/in situ ZnO hybrid, the characteristic diffraction peaks of the parent polymeric fibers as well as the peaks assigned to ZnO are clearly observed. Specifically, the strong diffraction peaks at 2 theta values of 31.95, 34.55, 36.35, 47.65, and 56.75 degrees correspond to the (100), (002), (101), (102), and (110) crystal planes of the hexagonal wurtzite ZnO structure (a = 0.315 nm and c = 0.529 nm, JCPDS Card No: 36-1451). Using the Debye–Scherrer equation and assuming that the line broadening was caused entirely by the particle size, the crystallite size of ZnO in chitin fibers based on the Gaussian fitted diffraction peak of (100) plane was found to be 11.7 nm, while the corresponding size derived from the peak of the (002) plane was 13.7 nm, suggesting that, on average, the sizes of the crystallites are of about 12 nm and marginally elongated along the c-axis. It has been appraised that the ZnO diffraction patterns can provide an evaluation of crystalline preferential growth, and thus of the average shape of crystallites, by taking into account the relative intensities of the (002) and (100) diffraction lines that are reflecting the crystallite axial ratio (c/a), i.e., the shape factor g [46,47,48]. In this context, aggregates of spherulitic morphology formed from single particles with c/a = 1 result in a (002) reflection intensity stronger than that of the (100), as is evident in our case, which indicates that the (nano)particles formed in the presence of nanofibers are approximately spherical. Finally, the characteristic peaks in both diffractograms at 2 theta values of 9.45, 12.85, 19.60, 22.40, and 26.65 degrees are attributed to the presence of chitin, as they correspond to the crystal planes (020), (101), (040) and (110), (130), and (013), respectively, of a-chitin crystallites [49].

The FTIR spectra of the ChNFs/in situ ZnO hybrid (Figure 4c) exhibit the characteristic peaks of the parent chitin polymer which is, in every respect, identical with that of a-chitin [49], given that ZnO does not absorb at the 4000–600 cm^−1^ region. Specifically, the two Amide I peaks at 1654 and 1623 cm^−1^ are characteristic of a-chitin crystallites. The first is assigned to hydrogen-bonded C=O with N-H groups of the same chain, while the second one is due to hydrogen bonding with the side chain CH_2_OH groups. Additionally, the OH and NH stretching modes in the 3600–3000 cm^−1^ region are also in line with the presence of a-chitin, in particular the NH stretching modes at 3260 and 3100 cm^−1^ and the OH stretching mode centered at ~3420 cm^−1^. Furthermore, the strong, broad band absorption observed extending below ~500 cm^−1^ in their spectrum is attributed to the presence of ZnO, whose Zn-O stretching modes are expected to be centered at about 410 cm^−1^ [47,50,51,52]. However, it is of interest to note that the position and number of Zn-O stretching bands depend on their particle morphology. The splitting of the absorption band takes place when going from the sphere to the cylinder, which can be utilized to delineate an average morphology of the ZnO particles [47]. Specifically, in the case of elongated wurtzite ZnO crystallites, the band splits into two maxima at around 512 and 406 cm^−1^ [46,47,50,51], while porous spherulitic specimens have been reported to give rise to two bands centered at 420 and 565 cm^−1^ [46,52]. The absence of these bands in our spectrum again points to the presence of sphere-like ZnO particles.

**Figure 5:**
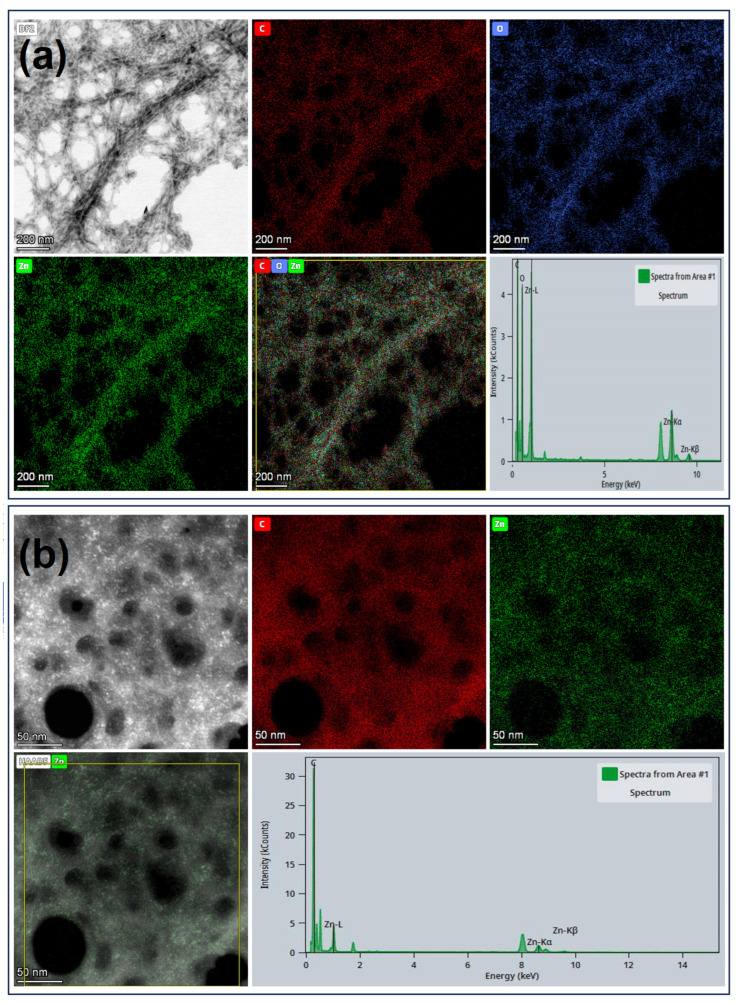
Scanning transmission electron microscopy (STEM) images of ChNFs/in situ ZnO nanofibers at two different magnifications and the corresponding energy-dispersive X-ray (EDX) elemental mapping images, as well as the corresponding EDX spectrum: carbon (red), zinc (green), and oxygen (blue), as well as their overlay. Scale bar length is 200 nm (**a**) and 50 nm (**b**).

TEM images (Figure 5) show both nanofiber bundles and nanofiber filaments with a thickness of a few nanometres. Although the ZnO nanoparticles are not clearly visible in these images, STEM elemental mapping clearly proves the presence of Zn along the bundles and filaments, as well as their colocalization with carbon and oxygen, verifying the presence of extremely small ZnO nanoparticles. Higher magnification images suggest that the size of these nanoparticles is less than 10 nm.

#### 3.2.2. ChNFs/Ex Situ ZnO Nanohybrid

For the synthesis of ChNFs/ex situ ZnO nanoparticles, we initially proceeded with the synthesis of ZnO nanoparticles employing the eco-friendly flame spray pyrolysis process, which at a second stage were allowed to electrostatically interact with the bio-derived chitin nanofibers with the aid of a sequential homogenization/sonication processes.

##### ZnO NPs

For the synthesis of ZnO nanoparticles, a one-step liquid flame spray pyrolysis (FSP) process was utilized. For the successful production of nanoparticles based on FSP, the obtention of suitable flame properties such as temperature, morphology, size and/or oxidative conditions are required. As evident in Section 2.4.1, certain parameters were exhaustively adjusted to ensure the production of nanoparticles having a monomodal size distribution.

More specifically, in order to reduce the residence time of the precursor mixture inside the flame, a feeding flow rate of 10–30 mL/min was employed, as it was found that the use of lower fluxes resulted in smaller flames. This resulted in smaller volumes available for particle growth and/or agglomeration, which favored the production of smaller nanoparticles. The gas flow was adjusted to 10–30 L/min which enabled a better control of the reaction due to a more stable flame morphology and temperature, thus avoiding potential temperature heating/cooling ramps. BET was carried out to determine the specific surface area and the average particle size of ZnO NPs [53]. It was found that ZnO NPs have a specific surface area of 34.8 m^2^/g and a mean particle size of 28 nm (Table S1).

**Figure 6:**
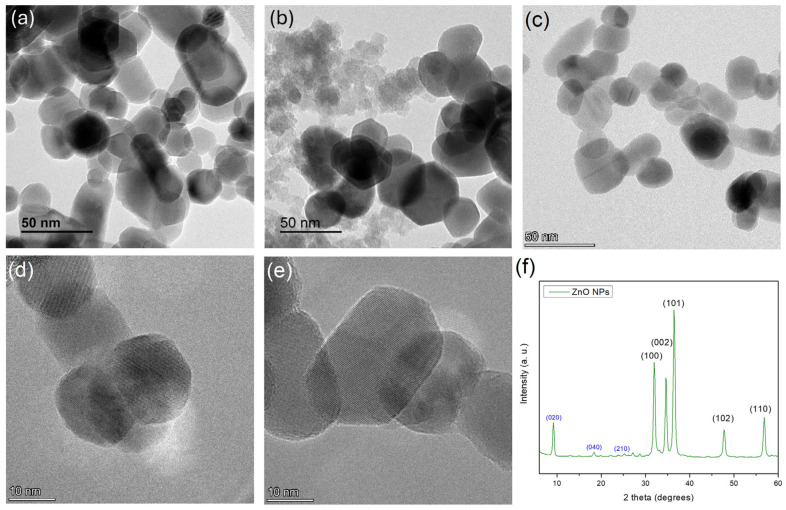
TEM images (**a**–**e**) and XRD patterns (**f**) of ZnO NPs synthesized by FSP.

TEM (Figure 6a–e) and SEM images (Figure S5) reveal the presence of crystalline nanoparticles of polyhedral plates and sizes around 20–50 nm (mean of 100 measurements using software), which are in full agreement with the mean particle size of ZnO NPs obtained from BET analysis. The X-ray diffractogram (Figure 6f) contains all the diffraction peaks that correspond to the crystal planes (100), (002), (101), (102), (110) of hexagonal wurtzite ZnO (JCPDS Card No: 36-1451), while no additional amorphous peaks were observed [54,55,56,57]. Apart from the major peaks, minor peaks are also evident at low angles, the most prominent of which are tentatively assigned to the (020), (040), and (210) planes of dibenzofuran corresponding to 2 theta values of 9.15, 18.39, and 23.64 degrees, respectively (JCPDS Card No: 36-1656). Indeed, recent studies have reported the presence of oxygenated polycyclic aromatic hydrocarbons in flame pyrolysis processes, which are generated in the fuel-rich region of the combustion chamber—the simplest being furan, and the larger ones being benzofuran and dibenzofuran [58,59]. Apparently, the higher molecular weight derivative (i.e., dibenzofuran) is not completely evaporated and remains in the final product. It should be noted that dibenzofuran is considered a relatively non-toxic compound [60], and therefore its traces are not expected to have any effect in our antibacterial and cytotoxicity results.

The particle size distribution of ZnO NPs aqueous dispersion (0.01 wt. %) was also studied using dynamic light scattering (DLS). As shown in Figure S6, a multimodal size distribution with three populations is observed. The first size distribution with a mean hydrodynamic value of 29 ± 3 nm is in line with the TEM measurements, while the second and third size distributions centered at ca. 90 and 400 nm are apparently the result of ZnO nanoparticle aggregates in pure water.

##### ChNFs/Ex Situ ZnO Preparation and Characterization

**Figure 7:**
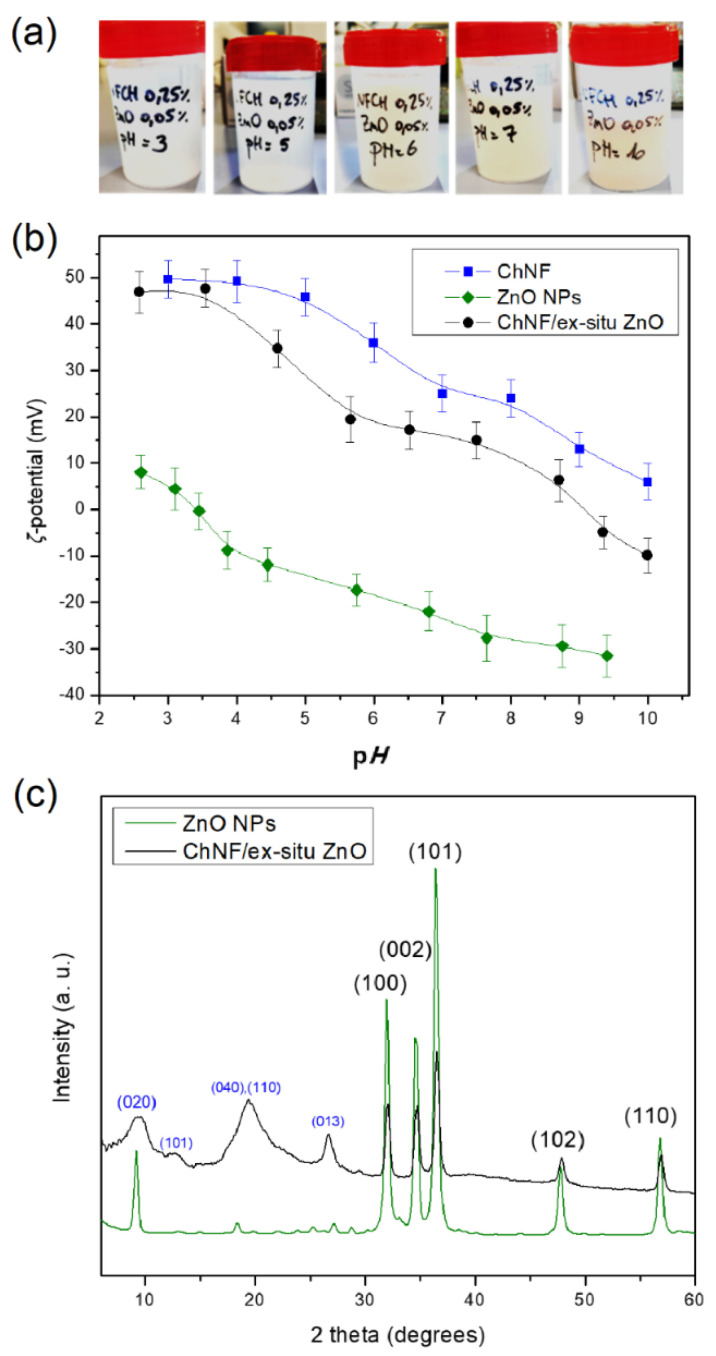
(**a**) Dispersion of ChNFs/ex situ ZnO hybrid (0.25% of ChNFs and 0.05% of ZnO) at different pH, after 48 h at rest; (**b**) ζ-potential of ZnO NPs, ChNFs and ChNFs/ex situ ZnO hybrid as a function of pH; (**c**) XRD patterns of ZnO NPs produced by FSP and of the corresponding ChNFs/ex situ ZnO nanohybrid.

Once the ChNFs and ZnO NPs had been prepared and characterized, the two components were mixed according to the process described in the Materials and Methods section, affording the ChNFs/ex situ ZnO nanohybrid (Figure 7a). It is known that nanoparticles with both acidic and basic functional groups at their external surface can undergo ionization when dispersed in aqueous media, and the degree of ionization and overall charge is influenced by the pH value of the medium. To this end, the *ζ*-potential values of both ZnO NPs and ChNFs dispersions in water were determined as a function of pH (Figure 7b). Given that the *ζ*-potential values of ZnO NPs and ChNFs dispersions at neutral pH are −23 mV and +25 mV, respectively, suggesting that both dispersions are stable and that their interaction would be rather strong, we decided to perform their interaction at this pH region by applying a homogenization/sonication step. The combination of the Silverson mixer and the Bran–son sonifier proved to be very effective, affording a very homogeneous suspension (Figure S7a). After 48h at rest, the agglomerates reappear (Figure S7b,c), but this agglomeration was completely reversible using the Silverson mixer. The aggregation sizes as revealed by DLS measurements were of about 1800 nm in radii (Figure S8), i.e., considerably increased compared to the respective DLS size of the parent chitin fibers.

The effect of pH on the stability of the ChNFs/ex situ ZnO hybrid was further studied as a function of pH values. Figure 7a shows that very stable dispersions are obtained at low pH values (2.5–6.5), which is verified by the corresponding *ζ*-potential values (Figure 7b) registered for ChNFs/ex situ ZnO (from +45 mV to +20 mV). Precipitation, however, occurs when the pH increases (pH > 6) (Figure 7a) as the *ζ*-potential values significantly reduce, approaching the isoelectric point (at pH ≈ 9), which can be correlated to the gradual deprotonation of the ChNFs amino groups. Moreover, the dispersions in the pH region between 3 and 5 are very transparent, which can be attributed to the partial solubilization of the nanohybrid in acidic media. Indeed, Cardoso et al. [61] showed that ZnO powder displayed faster kinetics of dissolution in the acidic medium.

**Figure 8:**
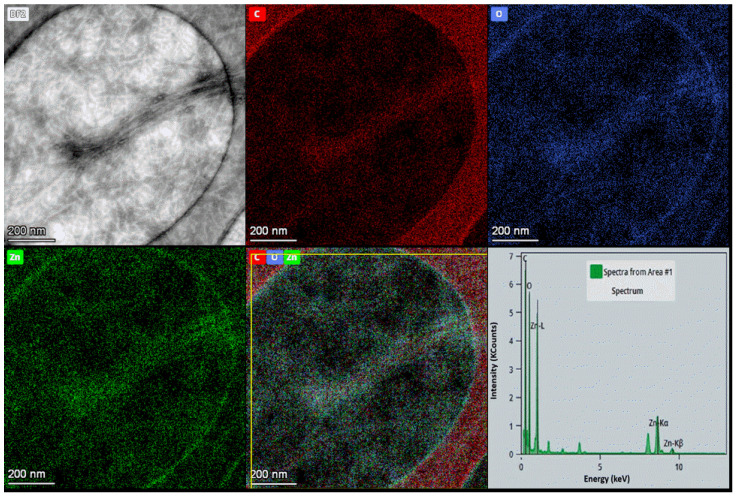
Scanning transmission electron microscopy (STEM) image of ChNFs/ex situ ZnO nanofibers and the corresponding energy-dispersive X-ray (EDX) elemental mapping images: C (red), Zn (green), and oxygen (blue), and their overlay (scale bar length is 200 nm) as well as the corresponding EDX spectrum.

The X-ray patterns of both the parent ZnO NPs and the ChNFs/ex situ ZnO hybrid after lyophilization are shown in Figure 7c. For the ChNFs/ex situ ZnO hybrid, the characteristic diffraction peaks of the parent polymer as exemplified above (Section 3.2.1.) as well as the diffraction peaks assigned to ZnO NPs, as previously discussed, are clearly observed, suggesting the presence of both components. This is also confirmed by TEM images and the corresponding energy-dispersive X-ray (EDX) mapping images (Figure 8). As it is shown in the bright field TEM image, bundles and filaments of ChNFs are clearly observed, while from the corresponding EDX elemental mapping images, the spatial distribution of carbon, oxygen, and zinc elements confirms the co-localization of zinc with carbon and oxygen atoms.

### 3.3. Cytocompatibility Studies of ChNFs/ZnO Nanohybrids

**Figure 9:**
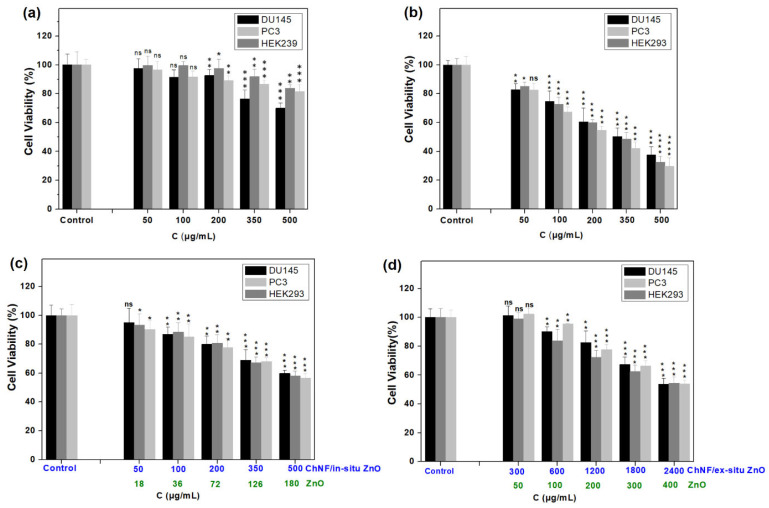
Comparative toxicities of ChNFs (**a**), ZnO NPs (**b**), ChNFs/in situ ZnO (**c**), and ChNFs/ex situ ZnO (**d**) on HEK293, DU145, and PC3 cells after incubation for 24 h as determined by MTT assays. Data are expressed as mean ± SD of six independent values obtained from at least three independent experiments. The statistical significance, obtained from Student’s paired two-tailed *t*-tests, follows the assignment: * *p* < 0.05, ** *p* < 0.01, *** *p* < 0.001, **** *p* < 0.0001, and ns, not significant (*p* > 0.5).

The utilization of ZnO NPs in various fields, including pharmaceutical, cosmetic, textile, and agricultural, has raised concerns about their toxicological effects on the environment and human health, highlighting the need to assess their cytotoxicity before their usage. In this context, the cytotoxicity of ChNFs/ex situ ZnO and ChNFs/in situ ZnO in comparison with that of ZnO NPs was studied against the normal human HEK293 kidney cell line, and the human DU145 and PC3 prostate cancer cell lines. Additionally, the cytotoxicity of ChNFs was also investigated under the same conditions. The cells were exposed to increasing concentrations of the nanomaterials and, following a 24 h incubation period, cell viability was evaluated, by measuring the mitochondrial redox function of cells employing the 3-(4,5-dimethylthiazol-2-yl)-2,5 diphenyltetrazolium bromide (MTT) assay. As shown in Figure 9, ChNFs were found to be non-toxic (survival: 75–80%) at the higher tested concentrations (500 μg/mL), while ZnO NPs decreased cell viability in all tested cell lines in a dose-dependent manner, with a maximum cytotoxicity at the highest tested concertation of 500 μg/mL (~30% cell survival). Similar cytotoxicity of ZnO NPs has also been reported in other publications [13,15,62], and attributed to the partial dissolution of ZnO NPs in lysosomes and late endosomes that have a pH value of about 5.5, where ZnO NPs are known to be partially soluble, resulting in the release of toxic zinc ions.

On the other hand, both ChNFs/ex situ and ChNFs/in situ ZnO hybrids showed improved cytotoxicity compared to the parent ZnO NPs, with ChNFs/ex situ ZnO being less toxic than ChNFs/in situ ZnO. Specifically, ChNFs/ex situ ZnO nanohybrid was found to be sub-toxic (cell survival ~60%) at the higher tested concentration that contained 400 μg/mL ZnO. On the other hand, ChNFs/in situ ZnO exhibited a comparative toxicity at the concentration of 500 μg/mL (corresponding to 180 μg/mL ZnO content). It is obvious that the presence of ChNFs reduced the toxicity of ZnO NPs, as chitin nanofibers are found to be non-toxic. Analogous results have been reported in cellulose-based composite films containing zinc oxide nanoparticles [11]. Moreover, chitin nanofibers may have a dual role either as a protective coating of ZnO NPs by inhibiting their degradation in lysosomes and late endosomes and/or as a scavenger of Zn (II) ions, as it known to form stable complexes with them [63,64,65].

### 3.4. Antibacterial Activity of ChNFs/ZnO Nanohybrids

The antibacterial properties of ChNFs/ex situ ZnO and ChNFs/in situ ZnO nanohybrids compared to ZnO NPs were evaluated on Gram (−) *E. coli* and Gram (+) *S. aureus* bacteria. In addition to these studies, the antibacterial activity of ChNFs was also investigated under the same conditions. Specifically, both bacterial strains were treated with various concentrations of ZnO NPs, ChNFs, or the ChNFs/ZnO hybrid materials, and then standard testing protocols M07-A9 and M26-A, published by the Clinical Laboratory Standards Institute (CLSI) [33,34], were employed for the determination of their minimum inhibitory concentration (MIC) and minimum bactericidal concentration (MBC) values against *E. coli* and *S. aureus* bacteria, respectively. The results are summarized in Table 1. As observed, ChNFs do not exhibit any activity against *E. coli* bacteria (MIC and MBC > 500 μg/mL), while they are active against *S. aureus* bacteria with MIC and MBC values equal to 100 μg/mL and 300 μg/mL, respectively. These findings are consistent with those presented in the literature, in which the antibacterial activity of chitin nanofibers is highly dependent on DDA and pH values. It was found that at pH and DDA values of 7 and approximately 30%, respectively, ChNFs do not show any antibacterial activity against *E. coli* bacteria, probably due to the presence of cationic amino groups [23]. In contrast, ChNFs were found to exhibit stronger antibacterial activity against Gram bacteria, particularly at low pHs [66]. Additionally, ZnO NPs showed a strong antibacterial effect towards both Gram (−) *E. coli* and Gram (+) *S. aureus* bacteria, with MIC and MBC values of 10–20 μg/mL and 50 μg/mL, respectively (Table 1). These results are expected given that the antibacterial activity of ZnO NPs is well known and depends on particle size, morphology, surface area to volume ratio and surface charge, concentration, preparation method, tested microorganisms, etc. [12,67,68,69,70,71,72]. Furthermore, both ZnO nanohybrids showed sufficient antibacterial properties, with ChNFs/in situ ZnO being more active against *E. coli* bacteria than ChNFs/ex situ ZnO. On the contrary, ChNFs/ex situ ZnO is more active against *S. aureus* than ChNFs/ex situ ZnO. Specifically, in the case of *E. coli* bacteria, the MIC and MBC values of ChNFs/in situ ZnO nanohybrid are 300 and 350 μg/mL (corresponding to 108 and 126 μg/mL ZnO, respectively), while for ChNFs/ex situ ZnO, both MIC and MBC values are 1600 μg/mL (200 μg/mL ZnO). On the contrary, for *S. aureus*, the MIC and MBC values of ChNFs/ex situ ZnO are 240 and 300 μg/mL (corresponding to 40 and 50 μg/mL ZnO), lower than those of the ChNFs/in situ ZnO nanohybrid, i.e., are 200 and 300 μg/mL (corresponding to 72 and 108 μg/mL ZnO). Comparing the antibacterial activities of nanohybrids with that of bare ZnO NPs, the latter showed slightly better activity than the ChNFs/in situ ZnO nanohybrid against both tested organisms. On the other hand, ZnO NPs exhibited marginally better activity than ChNFs/ex situ ZnO nanohybrid against *E. coli*, while they showed the same activity against *S. aureus* bacteria. Analogous results are presented in the literature. Composite membranes of chitin with 1% ZnO nanoparticles showed a substantial antibacterial activity, while pure chitin did not exhibit any antibacterial effect, which was attributed to the slow and sustainable release of Zn ions [73]. Similarly, the antibacterial properties of a chitin/Zn composite were lower than that of free Zn ions, while pure chitin acted as a nutrient for the bacteria [74]. On the other hand, a nanocomposite of chitin and ZnO NPs was found to enhance the overall antibacterial properties, suggesting that chitin might be contributing significantly to the antibacterial effects against Gram (+) strains [25]. Thus, although ZnO NPs showed slightly better antibacterial activity than both nanohybrids, the nanohybrids could be considered better antibacterial agents given that both are less toxic against mammalian cells compared to ZnO NPs, as previously demonstrated (Section 3.3). Herein, a balance between antibacterial activity and cytocompatibility was established, resulting in antibacterial nanomaterials with sufficient activity coupled with low toxicity.

To study the antibacterial mechanism of ZnO nanohybrids, two approaches were followed, i.e., (a) the determination of intracellular ROS production and (b) the morphological characterization of bacteria after treatment with ZnO nanohybrids.

**Figure 10:**
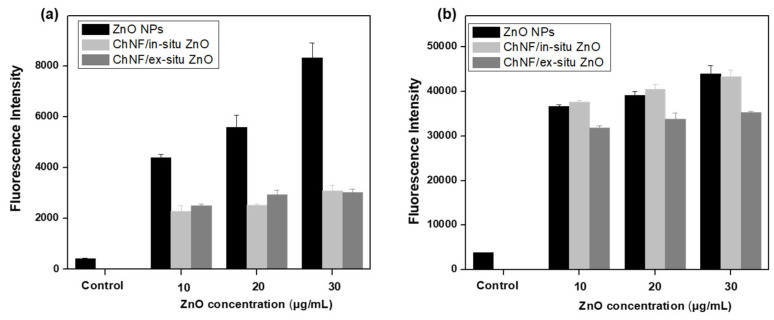
Intracellular reactive oxygen species production by ZnO NPs, ChNFs/in situ ZnO nanohybrid, or ChNFs/ex situ ZnO nanohybrid. (**a**) *E. coli* and (**b**) *S. aureus* bacteria were incubated with 10, 20, or 30 μg/mL ZnO NPs or 60, 120, or 180 μg/mL ChNFs/ex situ ZnO (corresponding to 10, 20, or 30 μg/mL ZnO, respectively) or 28.5, 57, or 85.5 μg/mL (corresponding to 10, 20 or 30 μg/mL ZnO, respectively) ChNFs/in situ ZnO for 5 h at 37 °C and the fluorescence intensity of the produced 2′,7′-dichlorofluorescein (DCF) was measured (λ_ex_ = 485 nm; λ_em_ = 530 nm). Untreated cells were used as control. Data are the means and SD of three independent experiments.

Oxidative stress causing the ROS production, such as superoxide anions (O_2_^−^), hydrogen peroxide (H_2_O_2_), and hydroxyl radicals (•OH), after bacteria treatment with various nanomaterials plays a significant role in both the survival and metabolic processes of bacterial cells, as ROS can cause damage to bacterial cell membranes as well as various cellular components, including DNA, proteins, and lipids [75,76,77]. Thus, intracellular ROS production was measured employing the 2′,7′-dichlorodihydrofluorescein diacetate (H_2_DCFDA) assay. H_2_DCFDA is a dye which can rapidly permeate bacterial cell membranes and is cleaved by intracellular esterases to form the cell-impermeable and non-fluorescent 2′,7′-dichlorodihydrofluorescein (DCFH). Then, DCFH interacts with the produced intracellular ROS, forming the highly fluorescent 2′,7′-dichlorofluorescein (DCF). Herein, bacteria after treatment with various concentration of ZnO NPs or nanohybrids were stained with H_2_DCFDA and the fluorescence intensity of the produced DCF was registered at 530 nm. It is evident (Figure 10) that both ZnO NPs and ZnO nanohybrids induce elevated ROS levels in both tested microorganisms, revealing that enhanced ROS production contributes to their antibacterial activities. ZnO NPs induce a higher ROS level in *E. coli* bacteria than both ZnO nanohybrids, confirming our hypothesis that ChNFs play a protecting role for ZnO NPs. On the contrary, ZnO NPs produce almost the same ROS level in *S. aureus* bacteria as both ZnO nanohybrids, justifying the similar antibacterial activities against this bacterial strain.

**Figure 11:**
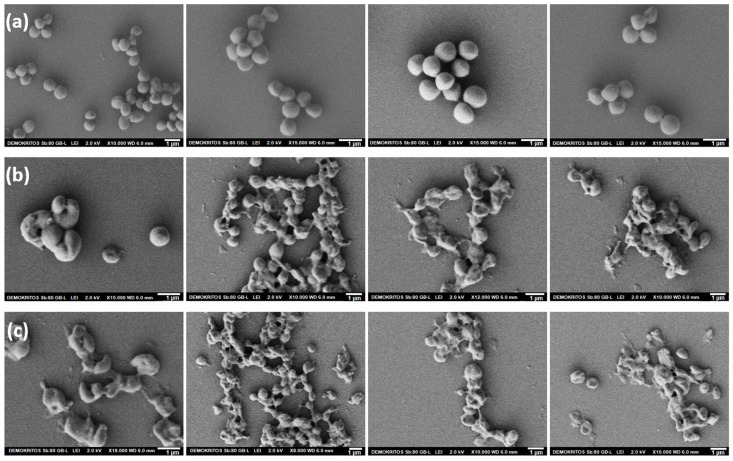
SEM images of *S. aureus* bacteria: untreated cells (**a**) as well as treated cells with ChNFs/in situ ZnO (**b**) and ChNFs/ex situ ZnO; (**c**) at ½MIC for 12 h at 37 °C.

Furthermore, the morphology of treated *S. aureus* was investigated by scanning electron microscopy (SEM). Specifically, cells were treated with ChNFs/in situ ZnO and ChNFs/ex situ ZnO at ½MIC for 12 h and then their surface morphology and the membrane structure changes were studied by SEM. In Figure 11, SEM images of the control (untreated *S. aureus* bacteria) and bacteria treated with ChNFs/in situ ZnO and ChNFs/ex situ ZnO were presented. As can be seen, the untreated bacteria (Figure 11a) have a typical spherical shape with intact cell walls, while after their exposure to nanohybrids (Figure 11b,c) considerable changes were observed in their morphology. Specifically, the bacteria appear to have lost their cellular integrity, as the cell walls appear wrinkled and damaged, coupled with leakage of intracellular contents and changes in cell shape, while many of them are dead. Similar results have been reported in the literature, where ZnO NPs at first cause the disruption of cell membranes and walls, followed by the leakage of intracellular contents, eventually leading to cell death. The mechanism of ZnO NPs’ antibacterial activity has not been fully elucidated [77,78,79], but it has been reported to be related to (a) the direct contact of ZnO NPs with bacteria, first causing the disruption of the cell membrane and then internalization into the cells either through bacterial membranes or endocytosis; (b) the production of reactive oxygen species (ROS), leading to enhanced cellular oxidative stress as well as to the inhibition of DNA replication and protein synthesis [77]; (c) the release of Zn^2+^ ions due to the dissolution of ZnO NPs, which cause the loss of cell membrane integrity as well as of the proton motive force, blocking the ion channels of the cell wall [80,81].

Herein, the ZnO nanohybrids may follow one of these mechanisms or a combination of them. The decrease in their antibacterial activity compared to bare ZnO NPs could be attributed to the presence of chitin, which prevents ZnO NPs from fast dissolution, thus controlling the release of Zn (II) ions as well as ROS production, which is evident in the respective mammalian cells experiments. Our results indicate that both in situ and ex situ nanohybrids exhibit satisfactory antibacterial activity, in the same concentration range in which no toxicity was observed against mammalian cells, and therefore they can be considered safe and effective antibacterial agents, better than bare ZnO NPs.

## 4. Conclusions

In this study, utilizing bioderived chitin and low-cost Zn inorganic precursors for the preparation of chitin nanofibers and ZnO nanoparticles, respectively, we successfully developed two non-toxic nanohybrid materials with improved aqueous stability and enhanced antibacterial properties. To this end, a simple chemical treatment of chitin derived from the biomass waste of shrimp shells with NaOH resulted in the formation of 30% of amino groups in chitin chains (deacetylation process), and the subsequent defibrillation process enabled the preparation of stable ChNFs aqueous dispersions. On the other hand, ZnO NPs were prepared by flame spray pyrolysis of an ethanolic zinc nitrate solution, affording nanoparticles of a polyhedral plate morphology with sizes around 20–50 nm. ChNFs/ZnO nanohybrids were prepared either by ensuring that the preformed ZnO NPs interact with the ChNFs (ex situ nanohybrid) under carefully chosen conditions, or by following a simple hydrothermal method for the in situ formation of ZnO nanoparticles of less than 10 nm in size using the ChNFs as templates (in situ nanohybrids). Both nanohybrids were physicochemically characterized by various methods for their size, morphology, charge, and stability. The ChNFs were found to efficiently stabilize the ZnO nanoparticles into the aqueous media. Their bactericidal properties were assessed against Gram (−) *E. coli* and Gram (+) *S. aureus* bacteria, while their cytocompatibility was assessed against mammalian cells. The obtained MIC values were found to range between 200 and 300 μg/mL corresponding to ca. 50–100 μg/mL of ZnO, while the cell viability at this concentration range was 80–100%, which is improved compared to the parent ZnO nanoparticles (cell viability 70%). Overall, this synthetic approach provides a balance between antibacterial activity and cytocompatibility, as both nanohybrids exhibited satisfactory antibacterial activity coupled with low cytotoxicity, indicating that they can be used as safe and effective antibacterial agents. Further in vivo studies proving their safety and their efficacy on antibiotic resistant bacteria are required for the validation of their potential as antibacterial surface coatings.

## Figures and Tables

**Table 1:** The MIC and MBC values of ZnO NPs, ChNFs, ChNFs/in situ ZnO and ChNFs/ex situ ZnO against *E. coli* and *S. aureus* bacteria. Both total concentrations and the corresponding ZnO actual concentrations are shown for the both nanohybrids.

		*E. coli*		*S. aureus*	
		MIC (μg/mL)	MBC (μg/mL)	MIC (μg/mL)	MBC (μg/mL)
**ZnO NPs**		10	50	20	50
**ChNFs**		>500	>500	100	300
**ChNFs/in situ ZnO**	Total ZnO	300 108	350 126	200 72	300 108
**ChNFs/ex situ ZnO**	Total ZnO	1200 200	1200 200	240 40	300 50

## Data Availability

The data presented in this study are available on request from the corresponding author.

## Supplementary Materials

The following supporting information can be downloaded at: https://www.mdpi.com/article/10.3390/nano15110809/s1, Figure S1: (a) FSP equipment, (b) flame, (c) ZnO NPs, (d) ZnO NP dispersion preparation, and (e) final ZnO NP aqueous suspension; Figure S2: (a) Chitin deacetylation reaction scheme, (b) FTIR spectra of the raw chitin and the deacetylated shrimp chitin (30% DDA), (c) UV–vis spectrum of deacetylated shrimp chitin film; Figure S3: Intensity-weighted hydrodynamic size distribution of a 0.25 wt. % ChNFs aqueous dispersion; Figure S4: Intensity-weighted hydrodynamic size distribution of a 1 mg/mL ChNFs/in situ ZnO nanohybrid aqueous dispersion; Figure S5: SEM images of ZnO NPs produced by FSP: (a) magnification ×10000, (b) magnification ×20000, and (c) magnification ×60000; Figure S6: Intensity-weighted hydrodynamic size distributions of a 0.01 wt. % ZnO NP aqueous dispersion; Figure S7: Dispersion of ChNFs/ex situ ZnO hybrid (0.25% of ChNFs and 0.05% of ZnO): (a) freshly prepared, (b) after 48h at rest, and (c) a magnified view of (b); Figure S8: Intensity-weighted hydrodynamic size distribution of ChNFs/ex situ ZnO nanohybrid aqueous dispersion; Table S1: BET results of ZnO NPs synthesized by FSP.

## Abbreviations

The following abbreviations are used in this manuscript:

BET specific surface area	Brunauer–Emmett–Teller specific surface area
ChNFs	Chitin nanofibers
CLSI	Clinical Laboratory Standards Institute
DDA	Degree of deacetylation
DLS	Dynamic Light Scattering
*E. coli*	Escherichia coli
EDTA	Ethylenediaminetetraacetic acid
EDX	Energy Dispersive X-ray Spectroscopy
FTIR	Fourier Transformed Infrared Spectroscopy
FSP	Flame Spray Pyrolysis
HPH	High-Pressure Homogenizer
H_2_DCFDA	2′,7′-dichlorodihydrofluorescein diacetate
MBC	Minimum bactericidal concentrations
MIC	Minimum inhibitory concentrations
MTT	3-(4,5-dimethylthiazol-2-yl)-2,5 diphenyltetrazolium bromide
NPs	Nanoparticles
NFs	Nanofibers
ROS	Reactive Oxygen Species
SEM	Scanning Electron Microscopy
TEM	Transmission Electron Microscopy
*S. aureus*	Staphylococcus Aureus
UV-vis	Ultraviolet–visible spectroscopy
XRD	X-Ray Diffraction
