# Supplementary material for: Sustainable Antibacterial Chitin Nanofiber/ZnO Nanohybrid Materials: Ex Situ and In Situ Synthesis, Characterization and Evaluation
Source: Nanomaterials (Basel). 2025 May 28;15(11):809. doi: 10.3390/nano15110809 (PMC12156430; doi:10.3390/nano15110809)
Supplement: Supplementary file 1 [file nanomaterials-15-00809-s001.zip › nanomaterials-3592425-supplementary.pdf]

Sustainable Antibacterial Chitin Nanofibers/ZnO Nanohybrid Materials: Ex-Situ and In-Situ Synthesis, Characterization and Evaluation

Caroline Piffet[1,*], Jean-Michel Thomassin[1], Emilie Stierlin[1], Job Tchoumtchoua[1], Claudio Fernández[2], Marta Mateo[2], Leyre Hernández[2], Kyriaki Marina Lyra[3], Aggeliki Papavasiliou[3], Elias Sakellis[3,4], Fotios K. Katsaros[3], Zili Sideratou[3], Dimitris Tsiourvas[3,*]

Supporting information

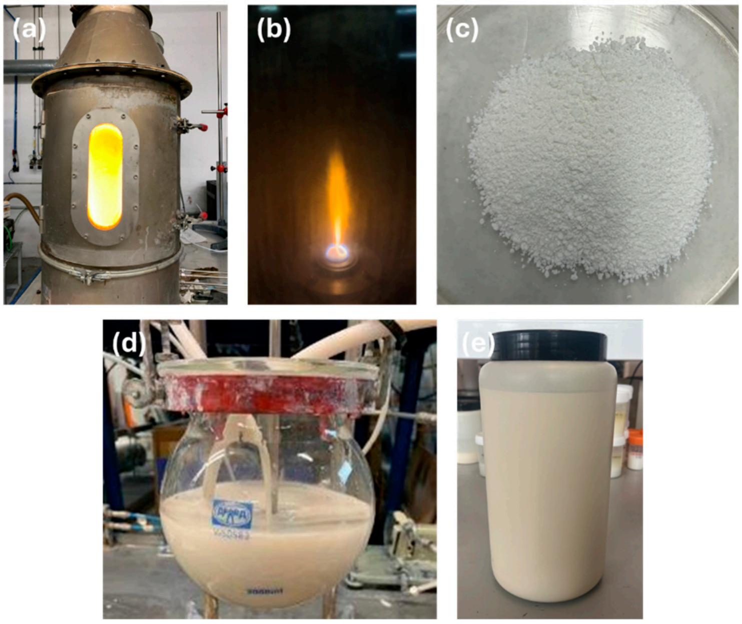

Figure S1. (**a**) FSP equipment, (**b**) flame, (**c**) ZnO NPs, (**d**) ZnO NPs dispersion preparation, and (**e**) final ZnO NPs aqueous suspension.

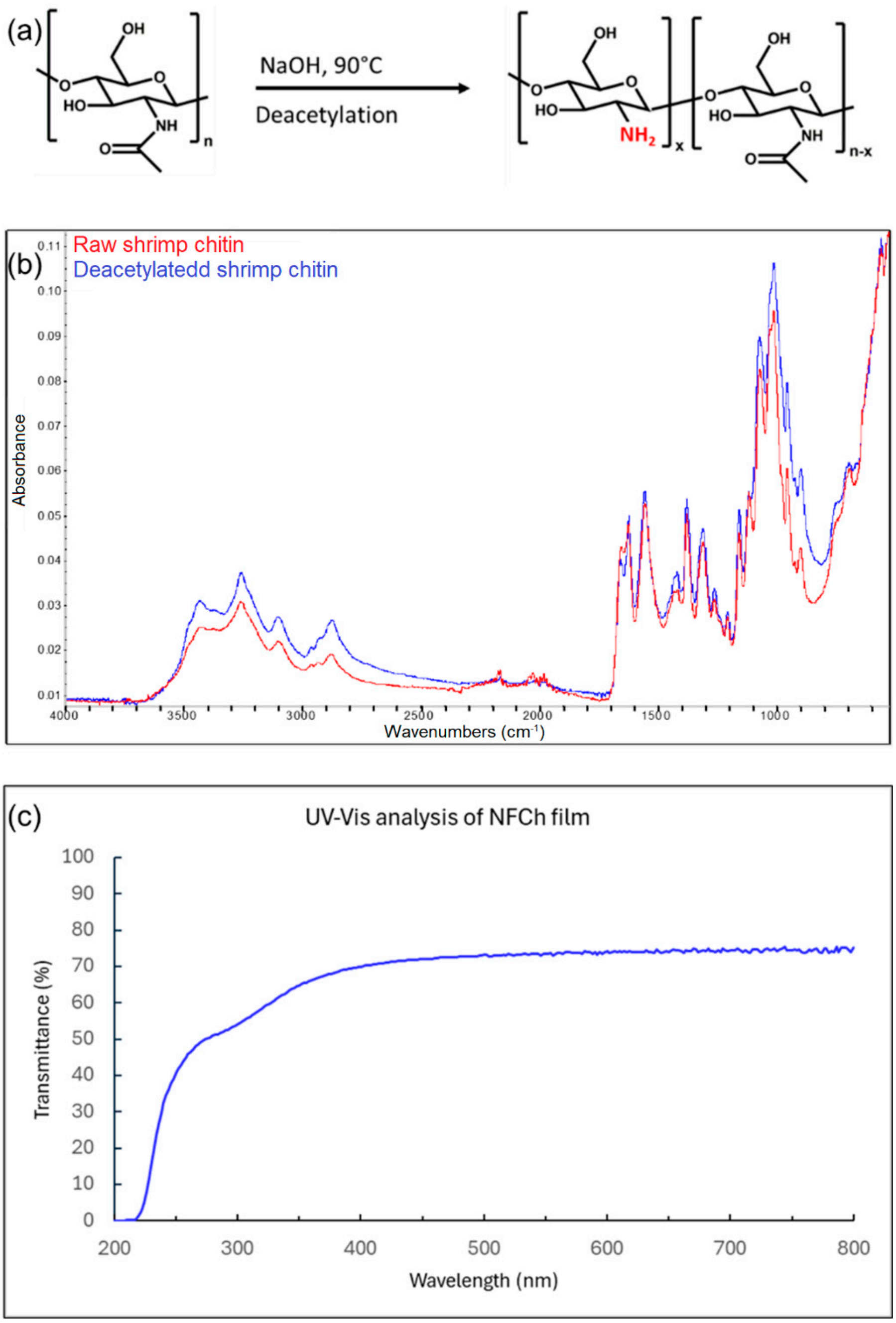

Figure S2. (**a**) Chitin deacetylation reaction scheme, (**b**) FTIR spectra of the raw chitin and the deacetylated shrimp chitin (30% DDA), (**c**) UV-vis spectrum of deacetylated shrimp chitin film.

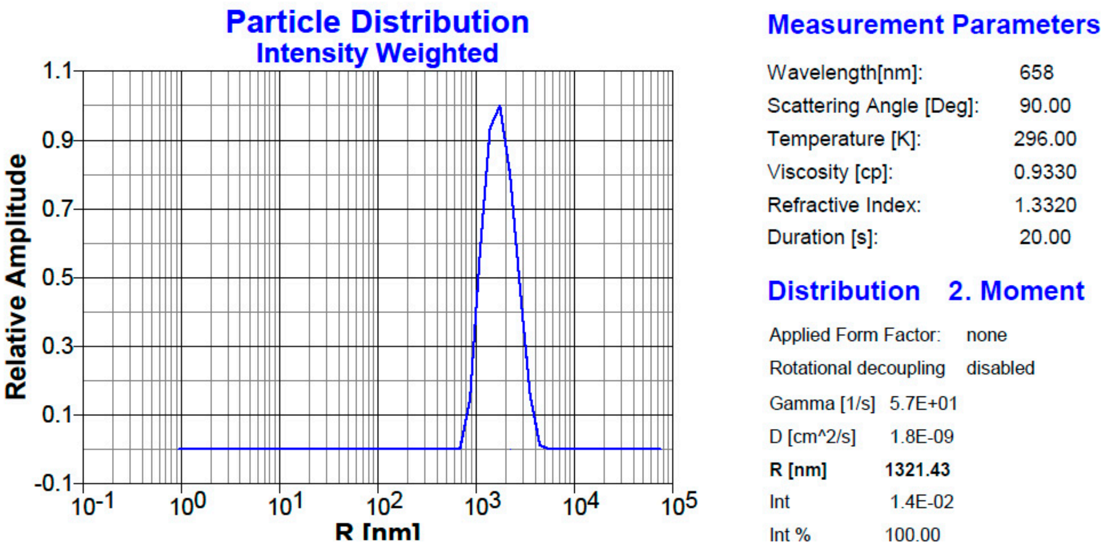

Figure S3. Intensity weighted hydrodynamic size distribution of a 0.25 wt. % ChNFs aqueous dispersion.

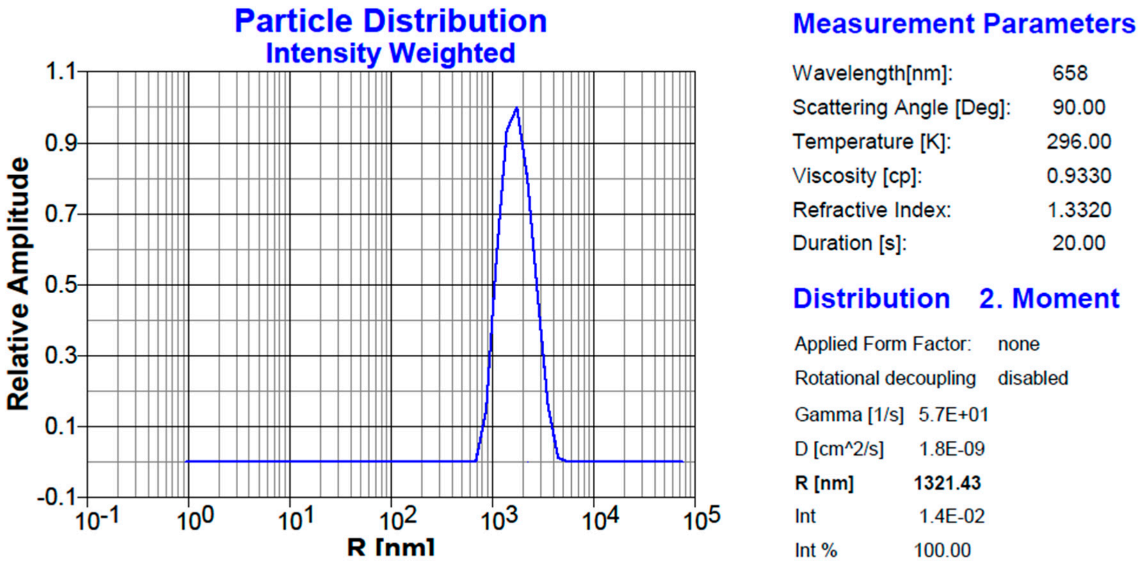

Figure S4. Intensity weighted hydrodynamic size distribution of a 1 mg/mL ChNFs/*in-situ* ZnO nanohybrids aqueous dispersion.

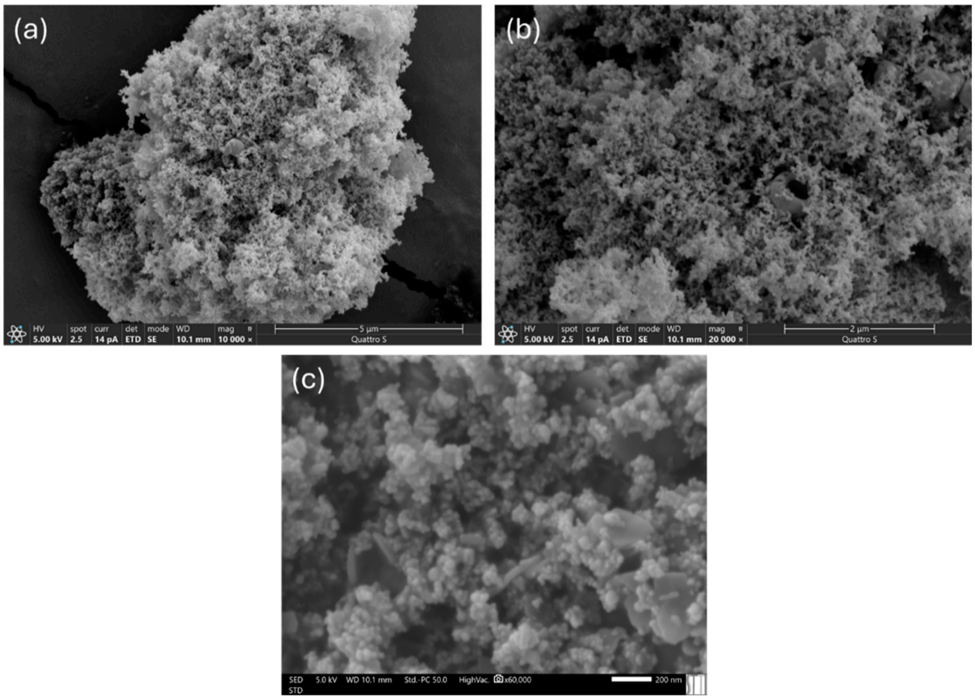

Figure S5. SEM images of ZnO NPs produced by FSP: (**a**) magnification ×10000, (**b**) magnification ×20000, and (**c**) magnification ×60000.

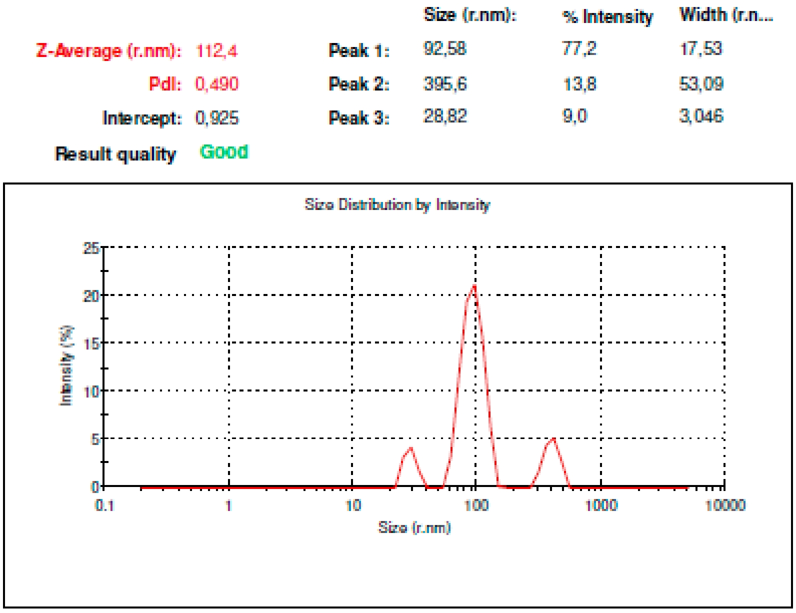

Figure S6. Intensity weighted hydrodynamic size distributions of a 0.01 wt. % ZnO NPs aqueous dispersion.

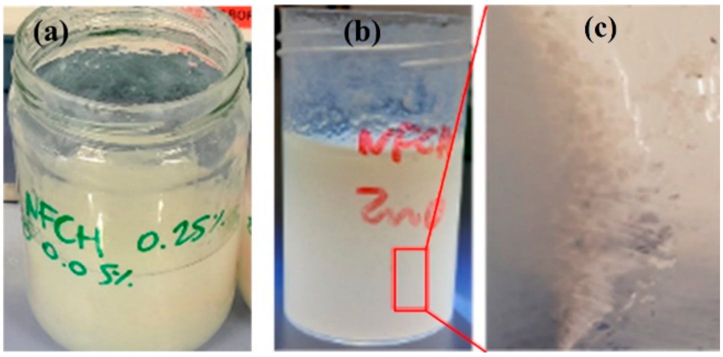

Figure S7. Dispersion of ChNFs/*ex-situ* ZnO hybrid (0.25% of ChNFs and 0.05% of ZnO): (**a**) Freshly prepared, (**b**) After 48h at rest, and (**c**) zoom of (**b**).

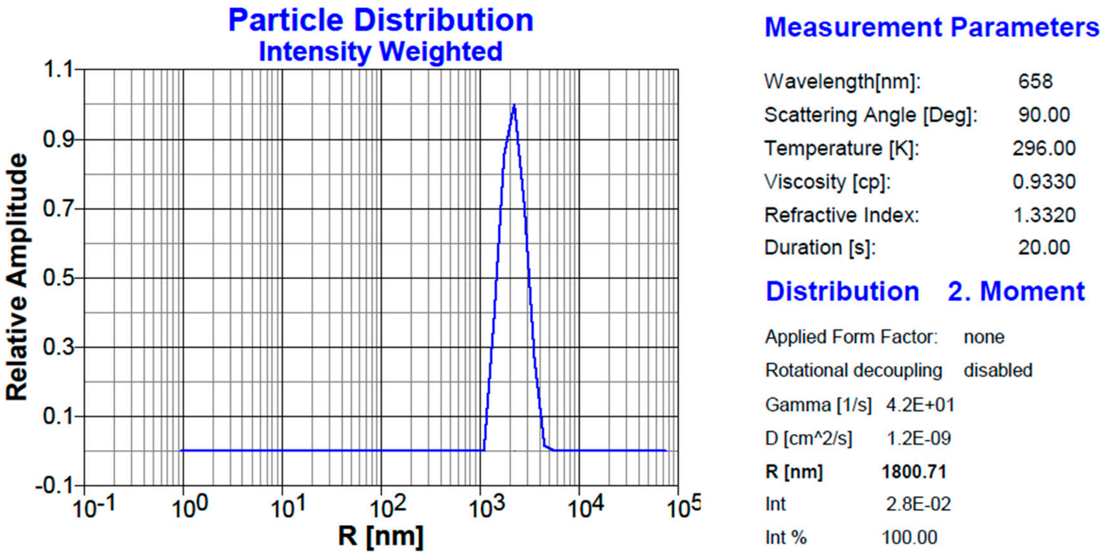

Figure S8. Intensity weighted hydrodynamic size distribution ChNFs/*ex-situ* ZnO nanohybrids aqueous dispersion (0.25% of ChNFs and 0.05% of ZnO).

Table S1. BET results of ZnO NPs synthesized by FSP.

BET surface Area Report		
BET Surface Area (mean ± SD)		34.8557 ± 0.1298 m^2/g
Slope (mean ± SD)		0.123956 ± 0.000459 g/cm^3 STP
Y-Intercept (mean ± SD)		0.000936 ± 0.000077 g/cm^3 STP
C		133.402293
Qm		8.0069 cm^3/g STP
Correlation Coefficient		0.9999794
Molecular Cross-Sectional Area		0.1620 nm^2
Relative Pressure (P/Po)	Quantity Adsorbed (cm^3/g STP)	1/[Q(Po/P - 1)]
0.055692393	6.4688	0.009117
0.099943958	7.2157	0.015389
0.150783452	7.9032	0.022466
0.201745550	8.5338	0.029616
0.252281425	9.1403	0.036914
0.302212777	9.7383	0.044474
